# I-κB kinase-ε deficiency improves doxorubicin-induced dilated cardiomyopathy by inhibiting the NF-κB pathway

**DOI:** 10.3389/fphys.2022.934899

**Published:** 2022-08-04

**Authors:** Yafeng Liu, Yueyue Xu, Yiwei Yao, Yide Cao, Ganyi Chen, Yuchen Cai, Wen Chen, Xin Chen, Zhibing Qiu

**Affiliations:** Department of Thoracic and Cardiovascular Surgery, Nanjing First Hospital, Nanjing Medical University, Nanjing, JS, China

**Keywords:** IKKε, doxorubicin, dilated cardiomyopathy, pyroptosis, connexin43

## Abstract

Dilated cardiomyopathy (DCM) can lead to heart expansion and severe heart failure, but its specific pathogenesis is still elusive. In many cardiovascular diseases, I-κB kinase-ε (IKKε) has been recognized as a pro-inflammatory molecule. In this study, wild-type mice (WT, *n* = 14) and IKKε knockout mice (IKKε-KO, *n* = 14) were intraperitoneally injected with a cumulative dose of 25 mg/kg with Dox or Saline five times in 30 days. Finally, the experimental mice were divided into WT + Saline group、WT + DOX group、IKKε-KO + Saline group and IKKε-KO + Dox group. Echocardiography was performed to assess cardiac structure and function. Moreover, the mechanism was validated by immunohistochemistry and western blotting. Our results demonstrated that compared to WT + Dox mice, IKKε-KO + Dox mice exhibited attenuation of dilated cardiomyopathy-related morphological changes and alleviation of heart failure. Additionally, compared to the WT mice after Dox-injected, the expression of fibrosis and proinflammatory were decreased in IKKε-KO mice, and the expression of cardiac gap junction proteins was much higher in IKKε-KO mice. Further testing found that pyroptosis and apoptosis in the myocardium were also ameliorated in IKKε-KO mice compared to WT mice after Dox was injected. Mechanistically, our results showed that deficiency of IKKε might inhibit the phosphorylation of IκBα, p65, RelB, and p100 in mouse heart tissues after Dox stimulation. In summary, our research suggests that IKKε might play an essential role in the development of Dox-induced dilated cardiomyopathy and may be a potential target for the treatment of dilated cardiomyopathy in the future.

## 1 Introduction

Dilated cardiomyopathy (DCM) is one of the most common causes of heart failure and the most number of heart transplants (Maron et al., 2006; Weintraub et al., 2017). Its etiology includes gene mutation, drugs, poisons, and alcohol, and the pathogenesis of dilated cardiomyopathy is unexplained. Although the causes of DCM vary, the phenotype and pathological characteristics of DCM are consistent (Schultheiss et al., 2019). Doxorubicin is a cancer chemotherapy agent whose dose-dependent cardiotoxicity has limited clinical use (Chang H. M. et al., 2017). This toxicity is of particular concern in patients with cancer susceptible to anthracyclines, such as breast cancer, many of whom die from heart failure (Singal and Iliskovic, 1998; Carvalho et al., 2009; Mehta et al., 2018). Moreover, an increasing number of studies have shown that Dox-induced cardiac pathology is similar to that of DCM (Kankeu et al., 2016; Wu et al., 2016; Xia et al., 2017).

IKKε (also known as IKK-inducible or IKK-i) was known as a non-canonical IKKs (Peters et al., 2000), which was involved in the regulation of many biological events including inflammatory responses, fibrosis, oncogenesis, apoptosis, and autophagy (Baldwin, 2012; Hsu et al., 2012; Patel et al., 2015; Zhou et al., 2019). Evidence has shown that IKKε can promote the phosphorylation of IκBα to activate the NF-κB signaling pathway (Shimada et al., 1999; Kravchenko et al., 2003; Solt and May, 2008). A previous study has suggested that IKKε can be activated by pro-inflammatory cytokines such as TNF-α(Tumor necrosis factor-α), IL-1β(Interleukin-1β), and IL-6(Interleukin-). Inhibiting IKKε could enhance the immunity of T cells to thwart tumor development and metastasis (Zhang et al., 2016). IKKε deficiency attenuated inflammation in Inflammatory Hyperalgesia by regulating the NF-κB pathway (Moser et al., 2011).

Activation of NF-κB–dependent transcription has been found in numerous heart diseases, including hypertrophic cardiomyopathy, myocardial infarction, ischemia/reperfusion injury, and so on (Jones et al., 2005; Hall et al., 2006; Baldwin, 2012; Kumar et al., 2012; Maier et al., 2012; Cao et al., 2021). In our previous studies, the role of IKKε in atherosclerotic lesions and aortic stenosis has been suggested (Cao et al., 2013; He et al., 2019). The IKKε-KO could attenuate mice’s pathological progression in Angiotensin II-Induced Myocardial Hypertrophy and aortic valve thickening (He et al., 2019; Cao et al., 2021). However, the role of IKKε in dilated cardiomyopathy is unclear. Herein, we aimed to investigate the potential role and molecular basis of IKKε in DOX-induced cardiotoxicity.

## 2 Materials and methods

### 2.1 Animals

The experiments on animals were performed to comply with the Institute of Laboratory Animal Research Guide for the Care and Use Laboratory Animals of the National Institutes of Health and approved by the Institutional Animal Care and Use Committee of Nanjing Medical University (Ethics Committee of Nanjing First Hospital). IKKε knockout mice (B6. Cg-Ikbketm1Tman/J (male; 8 weeks old; 22–27 g; *n* = 14) were obtained from the Jackson Laboratory (Bar Harbor, ME, United States) and rederived to achieve pathogen-free status in the Model Animal Research Center of Nanjing University (Nanjing, China). C57BL/6 mice (male; 8 weeks old; 22–27 g; *n* = 14) were netted from the Institutional Animal Care and Use Committee of Nanjing Medical University (Nanjing, China). All the mice were housed in specific pathogen-free box cages at room temperature, on a 12-h light/12-h dark cycle with free access to a regular diet and water.

### 2.2 Dox-induced mouse model of cardiotoxicity

As described in previous studies (Liu et al., 2020), the mice were intraperitoneally injected with a cumulative dose of 25 mg/kg doxorubicin (25316-40-9, Sigma-Aldrich) or saline *via* five times intraperitoneal injections (5 mg/kg i. p.) in 30 days. All the mice were divided into four groups, including the WT + Saline group, The WT + Dox group, the IKKε-KO + Saline group, and the IKKε-KO + Dox group. All the mice were housed in specific pathogen-free box cages at room temperature, on a 12-h light/12- h dark cycle with free access to a regular diet and water. The cardiac function was detected by echocardiography after the Dox injection. After the echocardiographic assessment, all mice were weighed and sacrificed under anesthesia, hearts were harvested immediately and heart weights were measured. Immediately after rinsing the heart in saline, protein and RNA sample extraction and dehydrated paraffin embedding were performed on another heart sample. The sample of protein and RNA was stored at −80°C.

### 2.3 Echocardiography evaluation

Mice were anesthetized with 1.5–2% isoflurane by inhalation and placed in supine position. Then, echocardiography was performed using a Vevo2100 ultrasound with a 30-MHz linear array ultrasound transducer (VisualSonic Inc., Toronto, Canada). Echocardiographic measurements were taken on M-mode to determine the left ventricular ejection fraction (LVEF), fractional shortening (FS), left ventricular end-diastolic diameter (LVEDd), and left ventricular end-systolic diameter (LVEDs) of each animal.

### 2.4 Western blotting analysis

Total protein samples were extracted from left ventricular tissue and 30 ug of protein separated by SDS-PAGE. Nuclear and cytoplasmic proteins were prepared from the cells using nuclear and cytoplasmic extraction reagent kits (Cayman Chemical, Ann Harbor, MI, United States) according to the manufacturer’s instructions. The proteins were transferred to polyvinylidene fluoride (PVDF) membranes (Millipore), washed third in Tris-buffered saline (TBS) with Tween diluted 1:1000 (TBST; Promega), for 10 min each time, then blocked with TBST containing 5% BSA for 1 h. The membranes were incubated with the following primary antibodies in TBST with Tween plus 5% BSA overnight at 4°C: anti-phosphorylated IKKε (1:1000, 3,416, CST), anti-Connexin43 (1:1000, ab11370, Abcam), anti-Bax (1:1000, 2772S, CST), anti-Caspase1 (1:1000, 2225T, CST), anti-Cleaved-Caspase3 (1:1000, 9661S, CST), anti-Caspase6 (1:1000, 9762T, CST), anti-Caspase9 (1:1000, 9508T, CST), anti-GSDMD (1:1000, ab219800, Abcam), anti-phosphorylated p65 (1:1000, cs3033, CST), anti-p65 (1:200; sc8008, Santa Cruz), anti-phosphorylated IκBα (1:1000, 2859s, CST), anti-IκBα (1:200; sc371, Santa Cruz), anti-phosphorylated p100/p52 (1:500, ab31474, Abcam), anti-p100/p52 (1:500, ab109440, Abcam), anti-phosphorylated RelB (1:500, ab47366, Abcam), anti-RelB (1:1000, ab180127, Abcam), HRP-conjugated Monoclonal Mouse Anti-glyceraldehyde-3-phosphate Dehydrogenase (GAPDH) (1:5000, KC-5G5, Kang Chen) and anti-Histone H3 (1:1000, ab1791, Abcam). The next day, the PVDF membranes were washed for 10 min each time with TBST three times. whereafter, the PVDF membranes were incubated with Goat Anti-Mouse IgG/HRP (1:5000, bs-0296G-HRP, Bioss) or anti-rabbit IgG, HRP-linked Antibody (1:5000, 7074P2, cell signaling technology) for 1 h at room temperature. Specific proteins were detected using an Immobilon Western chemiluminescent HRP substrate (WBKLS0500, Millipore) and captured on ChemiScope (3,300 Mini, Clinx Science Instruments). The mean gray value of each band was then semi-quantified with Chemi analysis software. All presented results are representative of at least three independent experiments.

### 2.5 Total RNA extraction and quantitative real-time PCR (q RT-PCR)

Total RNA was extracted from the left ventricle tissues using the TRIzol Reagent (Invitrogen, 15596-026). equal amounts of RNA (1 μg) were transformed into cDNA with the PrimScriptTM RT reagent Kit with gDNA Eraser (Takara, RR047A). Quantitative TaqMan PCR was conducted with SYBR Premix Ex TaqTM II (Takara, RR082A) by the Applied Biosystems 7,500 Real-Time PCR System. All data were normalized to GAPDH content and are expressed as fold increase relative to the expression level in a sham-operated control littermate mouse.

### 2.6 Histological and immunohistochemical staining and imaging

Mouse hearts were immediately immersed in 4% neutral phosphate-buffered paraformaldehyde (12 h), embedded in paraffin, and sectioned (4 μm). The sections of the specimens were evaluated under a light microscope after were stained with hematoxylin-eosin (HE), Masson’s trichrome, or wheat germ agglutinin (WGA) and were then observed for morphological changes and fibrosis in the myocardium.

For immunohistochemical staining, mouse heart tissues were gathered for morphological analysis with mice hearts prepared as 4-µm thick serial paraffin-embedded sections and rehydrated in graded alcohol. We treat the sections with 3% hydrogen peroxide for 15 min to block endogenous peroxidase activity and incubate them in imported goat serum (ZLI-9022, Beijing Zhongshan Biotechnology) to prevent nonspecific binding of the antibodies. The sections were then incubated separately for 14 h with antibodies against IKKε (1:100, 3,416, CST), connexin43 (1:100, ab11370, Abcam), IL-1β (1:50, sc7884, Santa Cruz), and IL-18 (1:100, ab71495, Abcam), and then with goat anti-rabbit or anti-mouse IgG (KIT-5004 and KIT-5001, MXB) for 1 h at 37°C in a humidified box. Each antibody’s signal was developed using the substrate diaminobenzidine (DAB, ZLI-9018, Beijing Zhongshan Biotechnology). The sections were counterstained with hematoxylin, and photomicrographs were taken with a Zeiss SCOPE. A1 camera. The immunohistochemistry results were analyzed based on Fromowitz semiquantitative analysis scores used to score the brown chromogen intensity (range: 0–7). The average score of each slice determined by two independent observers was used for later comparison.

### 2.7 TUNEL staining

Frozen mice ventricular tissues were cut into 4 μm‐thick sections and fixed in 4% paraformaldehyde at room temperature for 16 min. We performed the TUNEL assay according to the *in situ* apoptosis detection kit (Roche Diagnostics (Shanghai) Co., Ltd.). The sections are intubated with protease K for 20 min, followed by equilibration buffer for 30 min and TUNEL reaction mixture for 1 h in a dark humidified box at room temperature. The last, the sections were stained with Hoechst to label nuclei and examined using a fluorescence microscope. Only nuclei that were located in cardiac myocytes were considered.

### 2.8 Statistical analysis

The data of experiments are presented as the mean ± SE. Differences among groups were evaluated by an analysis of variance followed by a post hoc Tukey’s test. The two groups’ differences were assessed using Student’s t-test. All statistical analysis used SPSS software (version 17.0; SPSS, Inc.). A value of *p* < 0.05 was considered to indicate a statistically significant difference.

## 3 Result

### 3.1 I-κB kinase-ε was evaluated in mice with dilated cardiomyopathy

**FIGURE 1:**
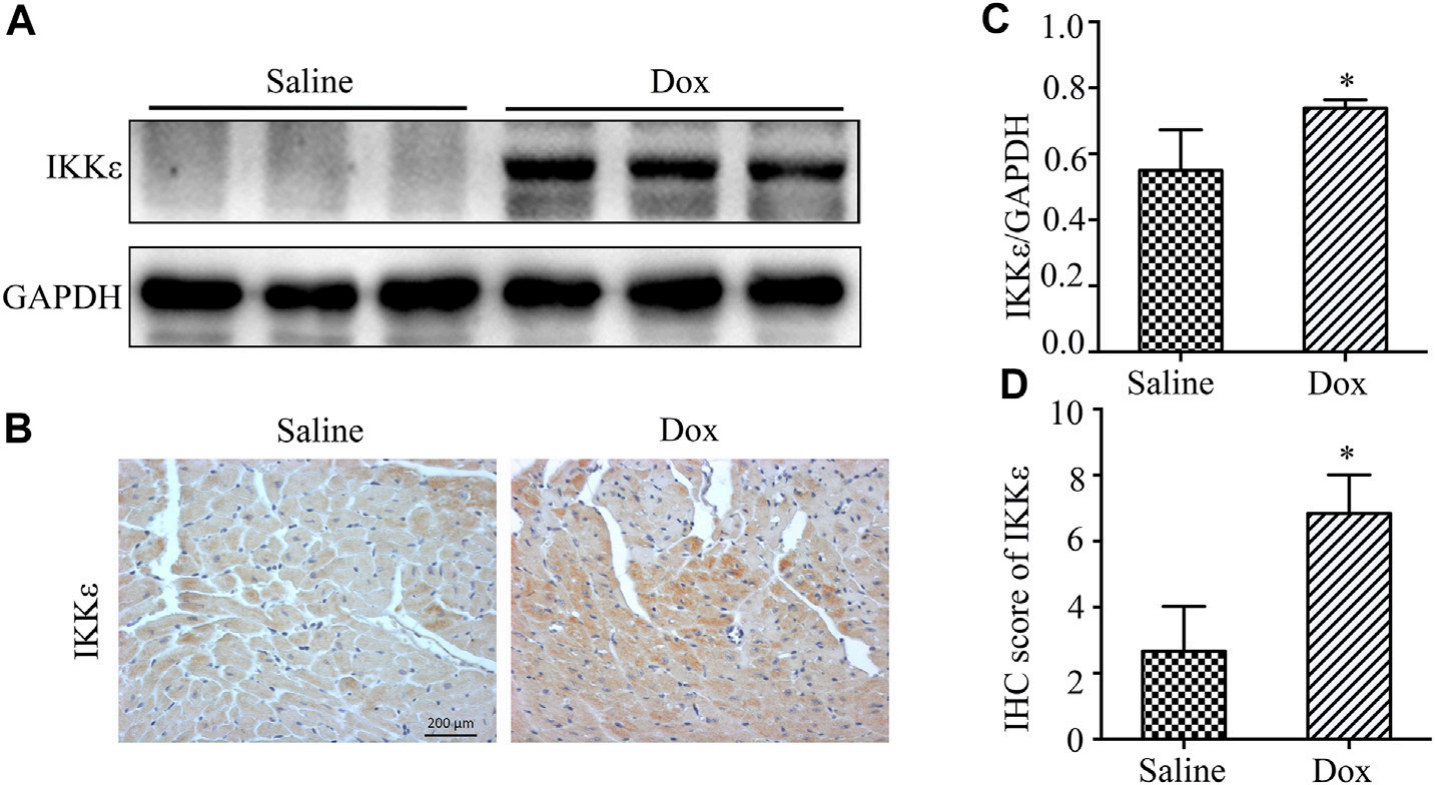
The expression of IKKε was increased in WT mice’ hearts after injection of Dox **(A)**. Representative western blot showing expression of IKKε in heart tissue after Dox injection (*n* = 4 mice per experimental group). **(B)**. Representative images of IHC staining of IKKε in WT mice’ hearts after Dox injection (*n* = 4 mice per experimental group, 400x; vs. Saline, *p < 0.05). **(C)**. Quantitative analyses of western blot of IKKε (vs. Saline, *p < 0.05). **(D)**. Quantitative analyses of IHC of IKKε (vs. Saline, *p < 0.05).

The western blotting analysis showed that the expression of IKKε was increased in the WT + Dox group compared with the WT + Saline group (Figure 1A, C). The images of IHC showed the same increasing expression of IKKε (Figure 1B, D). So, we found an apparent increasing expression of IKKε in the heart tissues of WT mice injected with Dox for 30 days compared to those of mice injected with saline.

### 3.2 I-κB kinase-ε knockout attenuated dox-induced cardiac dilatation and left ventricular dysfunction in mice

**FIGURE 2:**
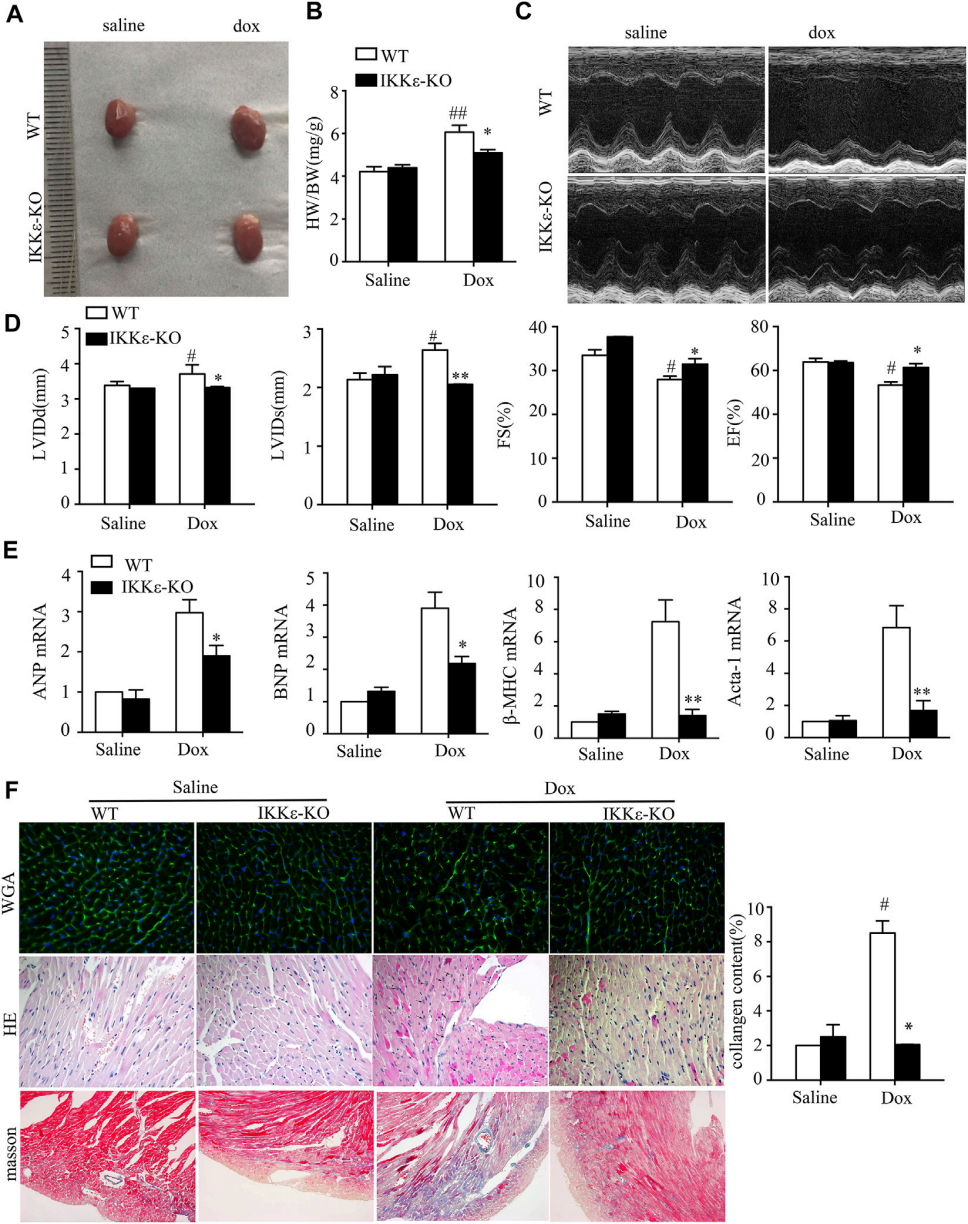
Deficiency of IKKε attenuated the development of Dox-induced dilated cardiomyopathy. **(A)**. Representative images of the hearts of WT and IKKε-KO mice. **(B)**. The ratio of heart weight/body weight between WT and IKKε-KO mice under Dox stimulation (*n* = 6 mice each group in HW/BW; vs. Saline or WT Dox, *p < 0.05) **(C,D)**. Representative images and parameters of echocardiography (LVIDd, LVIDs, FS, and EF) of WT and IKKε-KO mice injected with saline or Dox (*n* = 6 mice per experimental group; vs. Saline or WT Dox, *p < 0.05). **(E)**. The mRNA analysis of markers of heart failure (ANP, BNP, Acta-1, and β-MHC) in the heart of WT or IKKε-KO mice after Dox injection (*n* = 4 mice per experimental group; vs. Saline or WT Dox, *p < 0.05). **(F)**. Representative images of WGA staining, HE staining and Masson staining of WT and IKKε-KO mice (*n* = 4 mice per experimental group, 400x for HE and WGA staining; 200x for Masson). The small arrows in HE refer to the changed nuclei after doxorubicin stimulation. Analysis of collagen content of WT and IKKε-KO mice (*n* = 4 mice per experimental group, vs. Saline or WT Dox, *p < 0.05).

To examine the function of the IKKε in the development of DCM *in vivo,* we established a mouse model of DCM by intraperitoneally injecting Dox into WT and IKKε-KO mice. After Dox injection, WT mice’s hearts showed significant enlargement compared to Dox-induced IKKε-KO mice hearts (Figure 2A). The ratio of heart weight to body weight (HW/BW) among the four groups was evaluated, and the ratio in the WT + Dox group mice was higher than in the IKKε-KO + Dox group mice. (Figure 2B, Table 1). Left ventricular ejection fraction (LVEF) and fractional shortening (FS) were significantly lower in the WT + Dox group than in the IKKε-KO + Dox group mice. Moreover, left ventricular end-diastolic diameter (LVEDd) and left ventricular end-systolic diameter (LVEDs) were significantly increased in the WT + DOX mice compared to WT + Saline mice and IKKε+Saline mice, and this change was attenuated in IKKε-KO + Dox group mice (Figure 2C, D). The results of PCR showed that heart exhaustion markers, including atriopeptin (ANP), brain natriuretic peptide (BNP), β -cardiac myosin heavy chain (β-MHC), and skeletal muscle α-actin gene (Acta-1) significantly decreased in the IKKε-KO + Dox group mice when compared with the WT + Dox mice (Figure 2E). HE and WGA staining showed cardiomyocyte hypertrophy, myocardial structure destruction, and inflammatory cell infiltration in the WT + Dox group mice, but these were alleviated in the hearts of the IKKε-KO + Dox group mice (Figure 2F). Furthermore, Masson’s trichrome staining also showed that the collagen-stained area was remarkably larger in the WT + Dox group mice than in the IKKε-KO + Dox group mice (Figure 2F).

**TABLE 1:** heart weight and body weight of mice after Dox-induced.

number	Heart weight (mg)	Body weight(g)	HW/BW(mg/g)
WT + Saline	128 ± 2.966	31.167 ± 1.835	4.118 ± 0.235
IKKε+Saline	125.167 ± 4.665	30 ± 2.366	4.186 ± 0.234
WT + Dox	143 ± 4.648^#^	23.667 ± 1.366^#^	6.055 ± 0.4^#^
IKKε+Dox	130.167 ± 2.317^*^	26.333 ± 1.032^*^	4.94 ± 0.223^*^

^**#**^ *p* < 0.05 vs. WT + Saline,*p < 0.05 vs. WT + Dox.

### 3.3 I-κB kinase-ε knockout relieved fibrosis, inflammation, and destruction of gap junction structure after dox induction

**FIGURE 3:**
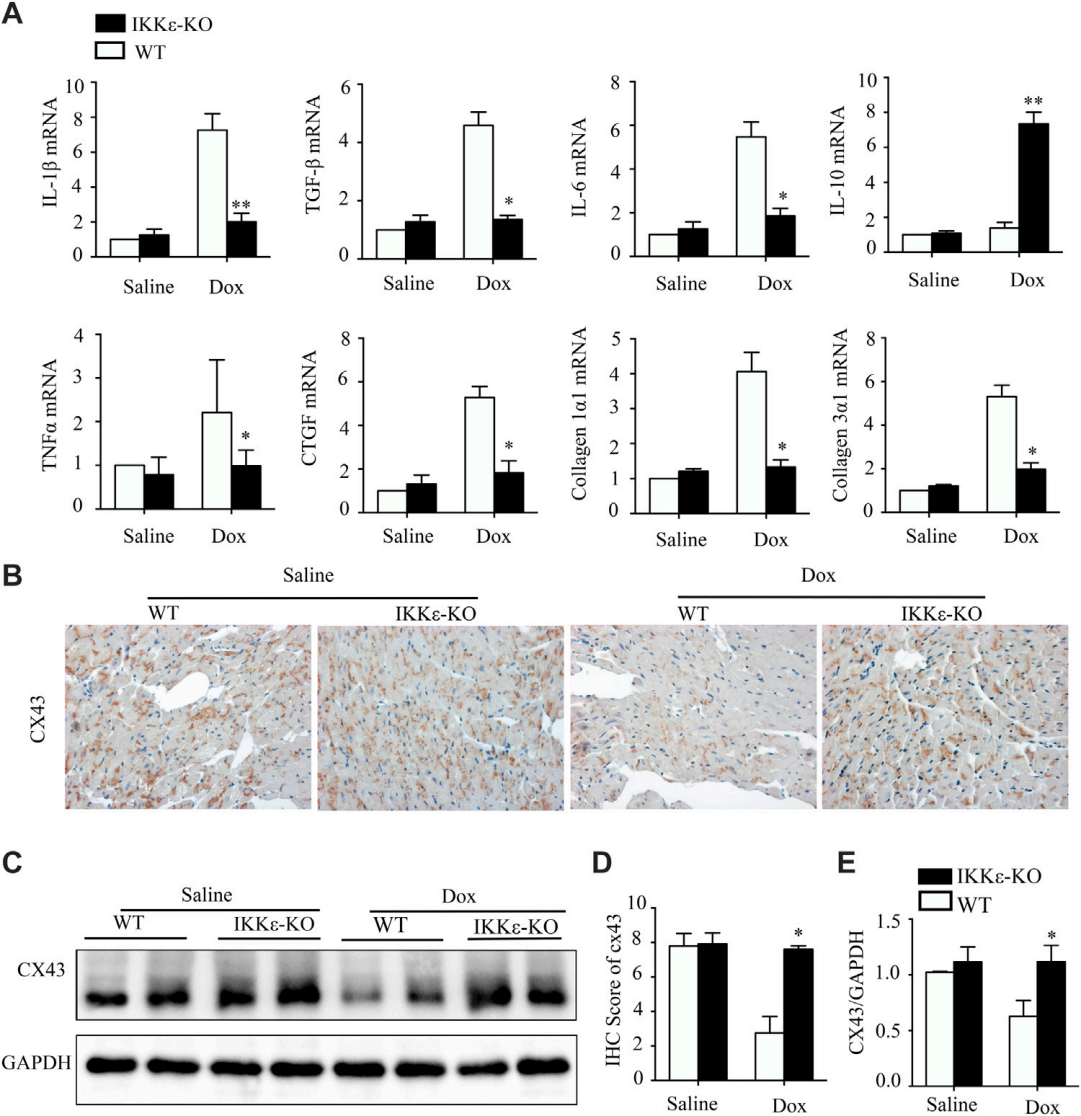
IKKε knockout relieved heart failure, fibrosis, inflammation, and destruction of gap junction structure after Dox induction. **(A)**. The mRNA analysis of markers of Inflammatory cytokines (IL-6, IL-1β, TNF-α, and IL-10), and collagen-related factors (CTGF, TGF-β1, Collagen 1a1, and Collagen 3a1) in the heart of WT or IKKε-KO mice after Dox injection (*n* = 4 mice per experimental group; vs. Saline or WT Dox, *p < 0.05). **(B,D)**. Representative IHC images and analyses of Cx43 in the heart tissues of WT and IKKε-KO mice after Dox stimulation (*n* = 4 mice per experimental group, 400x, vs. Saline or WT Dox, ^**#**^ *p*/*p < 0.05). **(C,E)** Representative western blot images and analysis of Cx43 in heart tissue after Dox injection (*n* = 4 mice per experimental group, ^**#**^vs. Saline or WT Dox, *p*/*p < 0.05).

Subsequently, we extensively examined the effect of IKKε on inflammation, and collagen deposition. The expression of proinflammatory factors (including TNF-α, IL-6, and IL-1β) and fibrosis markers (including CTGF, Collagen 1a1, and Collagen 3a1) showed the same trend in the two groups (Figure 3A). Furthermore, both western blots and IHC staining revealed that Cx43 expression significantly decreased in WT + Dox group mice compared with IKKε-KO + Dox group mice (Figures 3B–E). Thence, IKKε knockout might alleviate inflammation, collagen deposition, and destruction of gap junction structure after Dox induction.

### 3.4 I-κB kinase-ε knockout ameliorated pyroptosis and apoptosis in myocardial tissue after dox stimulation

**FIGURE 4:**
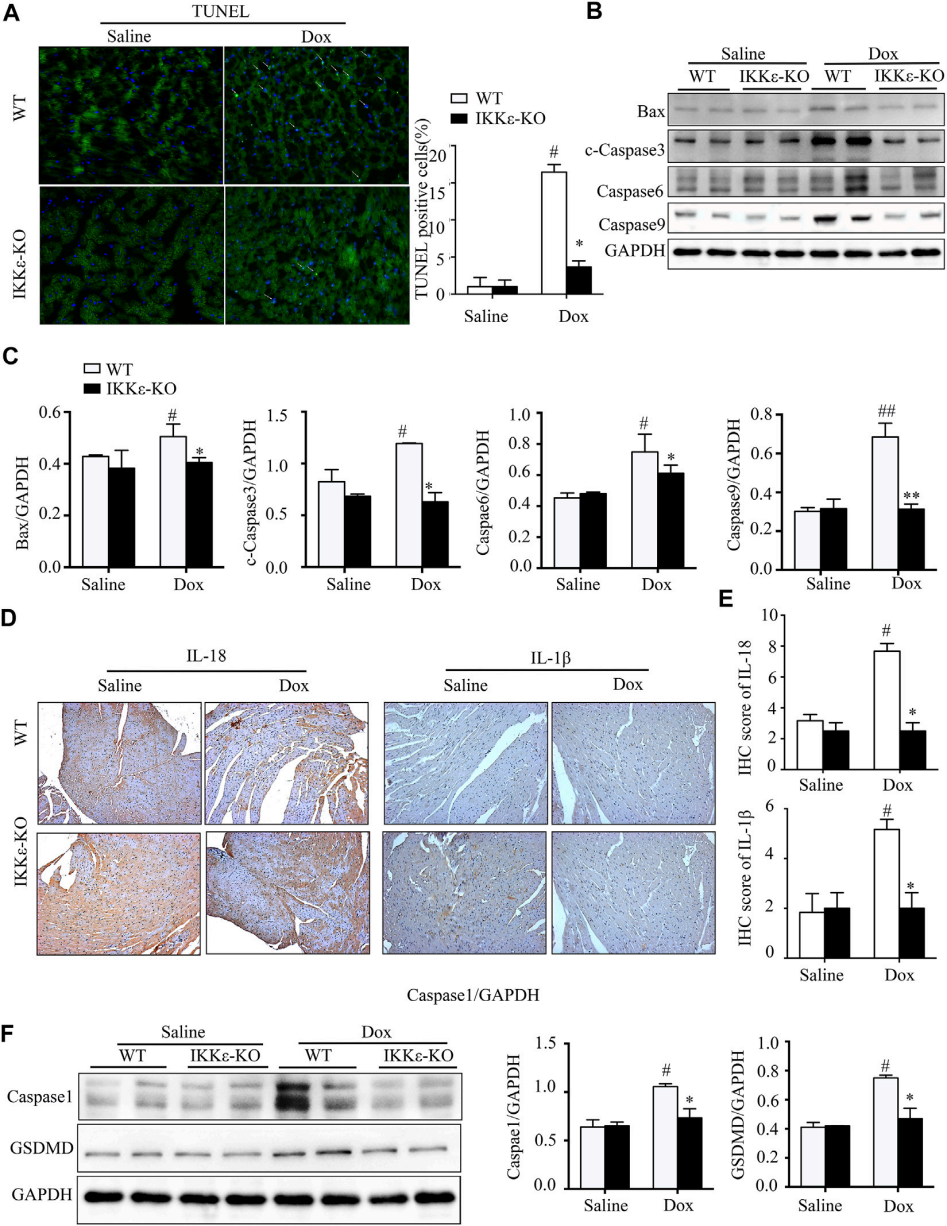
IKKε knockout ameliorated pyroptosis and apoptosis in myocardial tissue after Dox stimulation. **(A)**. Representative figures and analysis of TUNEL staining of tissue from WT and IKKε-KO mice after Dox injection (*n* = 4 mice per experimental group, vs. Saline or WT Dox, ^**#**^ *p*/*p < 0.05). **(B,C)**. The western blotting images and analysis of apoptosis-related proteins in the heart tissue of WT and IKKε-KO mice injected with Dox for 30 days (*n* = 4 mice per experimental group, vs. Saline or WT Dox, ^**#**^ *p*/*p < 0.05). **(D,E)**. Representative Immunohistochemistry images and analysis of IL-18 and IL-1β in the heart tissues of WT and IKKε-KO mice after Dox injection (*n* = 4 mice per experimental group, 200x, vs. Saline or WT Dox, ^**#**^ *p*/*p < 0.05). **(F)**. Representative western blot images and analysis of proteins associated with pyroptosis in heart tissue of WT and IKKε-KO mice injected with Dox for 30 days (*n* = 4 mice per experimental group, vs. Saline or WT Dox, ^**#**^ *p*/*p < 0.05).

The TUNEL result and the expression of apoptosis-marked proteins suggested that apoptosis was more severe in WT + Dox mice than in IKKε-KO + Dox mice (Figures 4A–C). Moreover, the expression levels of IL-1β and IL-18 were significantly higher in the WT + Dox group than in the IKKε-KO + Dox group, which were determined by IHC staining (Figure 4D, E). Additionally, western blot analysis showed the same tendency of caspase1 and GSDMD, which are the markers of pyroptosis, between the two groups (Figure 4F). In conclusion, the lack of IKKε might alleviate apoptosis and pyroptosis in Dox-induced DCM.

### 3.5 I-κB kinase-ε knockout inhibited the NF-κB signaling pathway in dox-induced dilated cardiomyopathy

**FIGURE 5:**
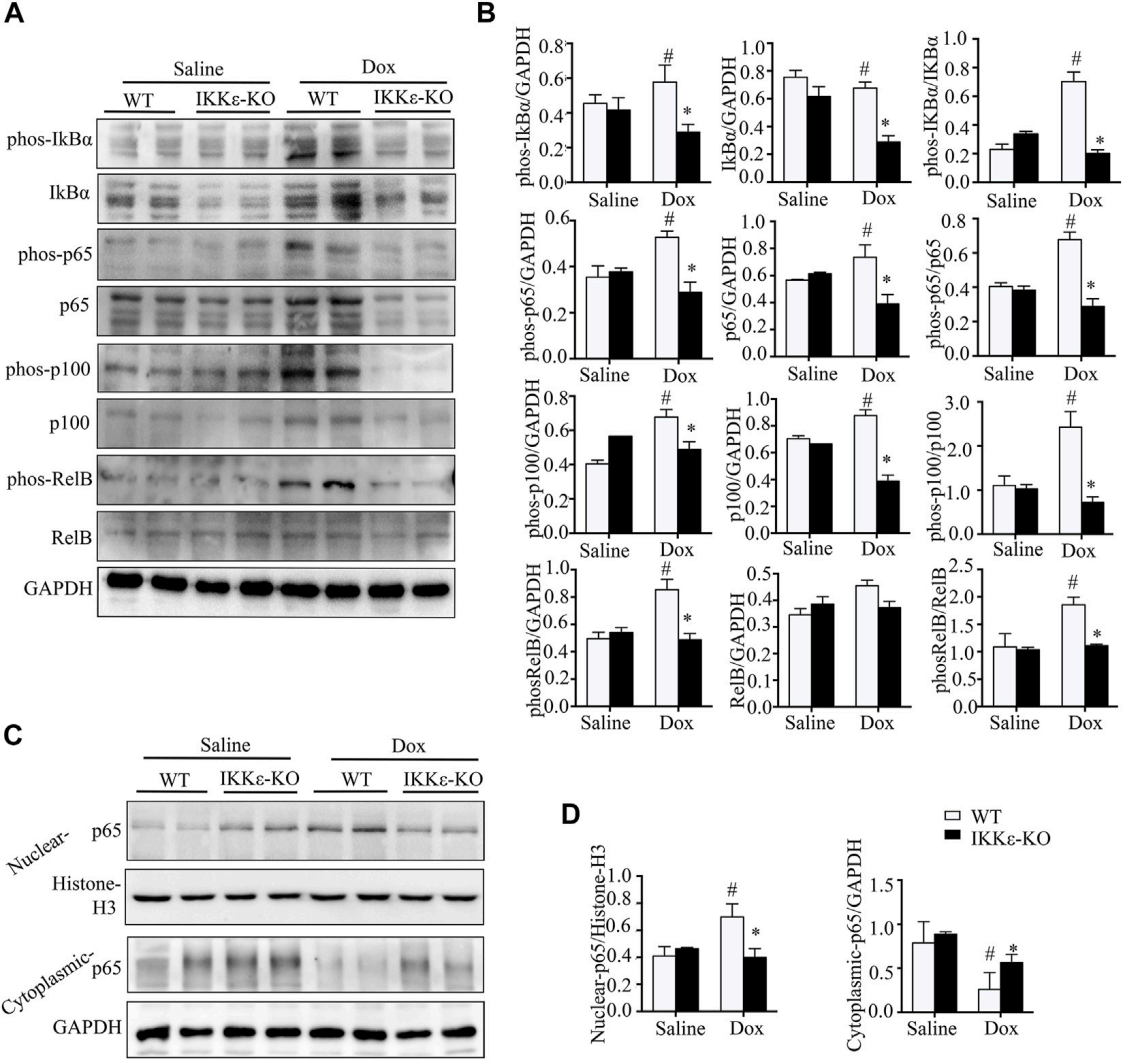
IKKε knockout inhibited the NF-κB signaling pathway in Dox-induced DCM. **(A)**. Representative western blots showing total protein and the phosphorylation levels of IκBα, RelB, p65, and p100 in the NF-κB pathway in heart tissues of WT and IKKε-KO mice injected with Dox (*n* = 6 mice per experiments). **(B)**. Quantitative analysis of western blotting of proteins related to the NF-κB pathway (*n* = 6 mice per experimental group; ^**#**^ *p*/*p < 0.05 vs. Saline or WT DOX). **(C)**. Representative western blots showing the nuclear and cytoplasmic protein levels of p65 in the NF-κB pathway in heart tissues from WT and IKKε-KO mice injected with Dox (*n* = 6 independent experiments). **(D)**. Relative quantitative analysis of nuclear translocation of p65 (*n* = 6 mice per experimental group; ^**#**^ *p*/*p < 0.05 vs. Saline or WT DOX).

Due to the significant differences in the inflammatory reaction, we evaluated the inflammation-related NF-κB signal pathways in WT and IKKε-KO mice’s hearts after Dox stimulation. Interestingly, we found apparent differences in IκBα, p65, RelB, and p100 in the NF-κB pathways between the two groups after intraperitoneal injection of Dox. The p-IκBα, p-P65, p-RelB, and p-p100 were higher expressed in the WT + Dox group compared with the IKKε-KO + Dox group (Figure 5A, B). Moreover, the nuclear translocation of p65 was increased in the WT + Dox group when compared to the IKKε-KO + Dox group (Figure 5C, D). The western blot results suggested that IKKε deficiency might inhibit the NF-κB signaling pathway in mouse hearts after Dox injection for 30 days.

## 4 Discussion

In this study, we demonstrated the role of IKKε on the development of DCM by intraperitoneal injection of doxorubicin in WT or IKKε-KO mice. Our study indicated that the knockout of IKKε alleviated Dox-induced cardiac dilatation and left ventricular dysfunction in mice. Moreover, the IKKε knockout protected the heart against inflammation, fibrosis, apoptosis, pyroptosis, destruction of gap junction structure, and pathological cardiac remodeling in response to long-term Dox stimulation. Thus, we provide the first time that IKKε might play a critical role in aggravating Dox-induced DCM.

Our previous study found that mice injected with doxorubicin showed pathophysiological changes related to dilated cardiomyopathy (Liu et al., 2020). In this study, the ratio of heart weight to body weight in the WT + Dox group was higher than that in the other groups. To examine whether IKKε knockout has cardioprotective effects in Dox-induced DCM, we examined murine cardiac function by echocardiography under steady-state conditions. Our echocardiographic data showed that the WT mice exhibited worst cardiac function with a lower EF and FS after Dox injection.

Moreover, echocardiographic examination revealed that the Dox-injected WT mice’s ventricular cavity was more extensive, and the ventricular wall was thinner than those of untreated WT mice. However, this change was alleviated in IKKε-KO + Dox group mice. As shown in Figure 3A, PCR also showed that heart failure markers (ANP, BNP, β-MHC, and Acta-1) were higher in the WT + Dox group than in the WT + Saline group. However, this deterioration of cardiac function was alleviated in the IKKε-KO + Dox group mice, which indicated a better cardiac function in IKKε-KO + Dox group mice compared to WT + Dox group mice.

Additionally, the IKKε is a non-canonical IκB kinase that plays a significant role in fibrosis and inflammation (Verhelst et al., 2013; Zhou et al., 2019). Previous studies (Cao et al., 2014; He et al., 2019) have shown that IKKε deficiency inhibits the inflammatory response and fibrosis in cardiovascular disease. IKKε knockout attenuates inflammation to promote cardiac protection in mice treated with a high-fat diet. Moreover, IKKε deficiency inhibits the fibrosis of cardiac remodeling and attenuated aortic valve thickening in apolipoprotein E deficient mice after angiotensin II-induced. As shown in Figure 2E, cardiomyocyte hypertrophy and inflammatory cell infiltration were evidenced in HE and WGA staining, which was alleviated in the hearts of IKKε-KO mice with Dox-induced. Moreover, Masson’s trichrome staining showed that the collagen area was remarkably larger in the WT + Dox group compared to IKKε-KO + Dox group mice. Consistent with previous studies (Corr et al., 2009; Bulek et al., 2011), we found an increased expression of anti-inflammatory factors (IL-10) and a decreased expression of proinflammatory factors (IL6, IL-1β, and TNF-α) in IKKε-KO mice after Dox injection. Therefore, IKKε knockout inhibits the inflammation during the process of Dox-induced DCM.

Dilated cardiomyopathy is often accompanied by arrhythmia. Reducing fibrosis is a primary therapeutic strategy because heart electrophysiology can be disrupted by fibrotic tissue and triggered life-threatening arrhythmias (Piek et al., 2019). Connexin43 is a cardiac gap junction protein that plays a vital role in the proper coordination of electrical conduction and mechanical contractility. Connexin 43 (Cx43), the most abundant cardiac gap junction protein, decreased in the decompensatory stage, or dilated cardiomyopathy might be associated with the destruction of gap junction structure (Kostin et al., 2003; Chang K. T. et al., 2017; Le Dour et al., 2017). In our study, the PCR analysis of fibrosis markers (CTGF, Collagen 1a1, and Collagen 3a1) showed that the IKKε-KO alleviated fibrosis in murine hearts after Dox stimulation. Additionally, the result of connexin43 tested by western blot and IHC staining suggested that destruction of cardiac gap junction structure was significantly attenuated in IKKε-deficient mice. Taken together, our results reveal that IKKε deficiency can reduce fibrosis and disruption of gap junction structures to protect the cardiac electrophysiological functions in Dox-induced DCM.

Numerous previous researches have revealed that apoptosis is associated with dilated cardiomyopathy (Zhang et al., 2017; Mazelin et al., 2016; Yin et al., 2022). Moreover, Dox increases ROS production in cardiomyocytes, which leads to mitochondrial damage and promotes apoptosis (Wu et al., 2016; Xia et al., 2017). The TUNEL staining and western blotting results of Bax, cleaved-caspase3, caspase6, and caspase9 showed a significantly higher expression of apoptosis in WT + Dox group mice than those of the group to IKKε-KO + Dox group mice, which suggested that the IKKε knockout could inhibit apoptosis in Dox-induced DCM. Pyroptosis is known as a form of programmed cell death, accompanied by inflammation. The characteristics of pyroptosis are disruption of the plasma membrane and release of cellular contents and proinflammatory mediators, including IL‐1β and IL‐18 (Ge et al., 2018).

Moreover, pyroptosis plays a role in many cardiovascular diseases, including atherosclerosis, heart failure, and cardiomyopathy (Vande Walle and Lamkanfi, 2016). Gasdermin-D (GSDMD) is known as the critical executioner of pyroptosis (Vande Walle and Lamkanfi, 2016). In addition, a previous study suggested that NF-kB was an essential transcription factor of GSDMD (Liu et al., 2017). Promoting the phosphorylation of the NF-κB subunit p65 increases the production and release of IL‐1β(Denkers et al., 152019). We detected the pyroptosis-related proteins and found that the expression of representative factors of pyroptosis (IL-1β, IL-18, caspase1, and GSDMD) was higher in WT + Dox mice than in IKKε-KO + Dox mice. Consequently, deficiency of IKKε could attenuate apoptosis and pyroptosis in Dox-induced DCM.

IKKε is a member of the IKK complex, which regulates the NF-κB pathway. Numerous previous studies have verified that IKKε is associated with phosphorylation of p65 in the classical NF-κB pathway (Moser et al., 2011; Yi et al., 2013; Changchun et al., 2014; Yang et al., 2018). In our study, the western blotting showed that the phosphorylation of IκBα, p65, RelB, and p100 was increased in WT mice after Dox injection. However, this trend was not found in Dox-treated IKKε knockout mice; and the nuclear translocation of p65 which is a significant member of the NF-κB pathway was inhibited by IKKε knockout in Dox-induced mice. Thus, we hypothesized that IKKε has a relationship with the NF-κB pathway in Dox-induced murine DCM.

The Dox-induced DCM in mice occurs mainly through the direct lesion of cardiomyocytes, leading to inflammation, cardiomyocyte apoptosis, and pyroptosis. Although this model is similar to human DCM, it does not fully simulate many human DCM aspects. Limited by time, this study only performed the research *in vivo,* which revealed IKKε as an essential regulator in Dox-induced DCM development. In the future, we will clarify the exact mechanism of IKKε in Dox-induced rat cardiomyocytes *in vitro*.

In conclusion, IKKε-KO attenuates Dox-induced DCM in. mice and reduces the inflammatory reaction, apoptosis, pyroptosis, and destruction of gap junction structure by inhibiting the NF-κB pathway. Therefore, our study might find a novel therapeutic target for the treatment of DCM.

## Data Availability

The original contributions presented in the study are included in the article/Supplementary Material, further inquiries can be directed to the corresponding authors.

## References

[B1] Baldwin A. S. (2012). Regulation of cell death and autophagy by IKK and NF-κB- critical. mechanisms in immune function and cancer. Immunol. Rev. 246, 327–345. 10.1111/j.1600-065X.2012.01095.x 22435564

[B2] Bulek K. Liu C. Swaidani S. Wang L. Page R. C. Gulen M. F. (2011). The inducible kinase IKKi is required for IL-17-dependent signaling associated with neutrophilia and pulmonary inflammation. Nat. Immunol. 12, 844–852. 10.1038/ni.2080 21822257 PMC3282992

[B3] Cao C. Zhu Y. Chen W. Li L. Qi Y. Wang X. (2013). IKKε knockout prevents high fat diet induced arterial atherosclerosis and NF-κB signaling in mice. PLoS One 8, e64930. 10.1371/journal.pone.0064930 23741427 PMC3669140

[B4] Cao C. Li L. Chen W. Zhu Y. Qi Y. Wang X. (2014). Deficiency of IKKε inhibits inflammation and induces cardiac protection in high-fat diet-induced obesity in mice. Int. J. Mol. Med. 34, 244–252. 10.3892/ijmm.2014.1746 24789209

[B5] Cao Y. Li L. Liu Y. Chen G. Tao Z. Wang R. (2021). I-κB kinase-ε deficiency attenuates the development of angiotensin II-induced myocardial hypertrophy in mice. Oxid. Med. Cell. Longev. 2021, 6429197. 10.1155/2021/6429197 33628362 PMC7886514

[B6] Carvalho C. Santos R. X. Cardoso S. Correia S. Oliveira P. J. Santos M. S. (2009). Doxorubicin: the good, the bad and the ugly effect. Curr. Med. Chem. 16, 3267–3285. 10.2174/092986709788803312 19548866

[B7] Chang H. M. Moudgil R. Scarabelli T. Okwuosa T. M. Yeh E. T. H. (2017). Cardiovascular complications of cancer therapy: Best practices in Diagnosis, prevention, and management: Part 1. J. Am. Coll. Cardiol. 70, 2536–2551. 10.1016/j.jacc.2017.09.1096 29145954 PMC5825187

[B8] Chang K. T. Cheng C. F. King P. C. Liu S. Y. Wang G. S. (2017). CELF1 mediates connexin 43 mRNA degradation in dilated cardiomyopathy. Circ. Res. 121, 1140–1152. 10.1161/CIRCRESAHA.117.311281 28874395

[B9] Changchun C. Liangpeng L. Wen C. Zhu Y. Qi Y. Wang X. (2014). Deficiency of IKKε inhibits inflammation and induces cardiac protection in high-fat diet-induced obesity in mice. Int. J. Mol. Med. 34, 244–252. 10.3892/ijmm.2014.1746 24789209

[B10] Corr M. Boyle D. L. Ronacher L. Flores N. Firestein G. S. (2009). Synergistic benefit in inflammatory arthritis by targeting I kappaB kinase epsilon and interferon beta. Ann. Rheum. Dis. 68, 257–263. 10.1136/ard.2008.095356 18653628 PMC2713581

[B11] Denkers E. Y. Pandori W. J. Lima T. S. Kao T. H. Gov L. Lodoen M. B. (1520). Toxoplasma gondii activates a Syk-CARD9-NF-κB signaling axis and gasdermin D-independent release of IL-1β during infection of primary human monocytes. PLoS Pathog. 15, e1007923. 10.1371/journal.ppat.1007923 PMC6730955 31449558

[B12] Ge X. Li W. Huang S. Yin Z. Xu X. Chen F. (2018). The pathological role of NLRs and AIM2 inflammasome-mediated pyroptosis in damaged blood-brain barrier after traumatic brain injury. Brain Res. 1697, 10–20. 10.1016/j.brainres.2018.06.008 29886252

[B13] Hall G. Hasday J. D. Rogers T. B. (2006). Regulating the regulator: NF-kappaB signaling in heart. J. Mol. Cell. Cardiol. 41, 580–591. 10.1016/j.yjmcc.2006.07.006 16949095

[B14] He S. Nian F. Chen W. Yin L. Auchoybur M. L. Tao Z. (2019). I-κB kinase-ε knockout protects against angiotensin II induced aortic valve thickening in apolipoprotein E deficient mice. Biomed. Pharmacother. 109, 1287–1295. 10.1016/j.biopha.2018.10.083 30463808

[B15] Hsu S. Kim M. Hernandez L. Grajales V. Noonan A. Anver M. (2012). IKK-epsilon coordinates invasion and metastasis of ovarian cancer. Cancer Res. 72, 5494–5504. 10.1158/0008-5472.CAN-11-3993 22942254 PMC3488159

[B16] Jones W. K. Brown M. Wilhide M. He S. Ren X. (2005). NF-kappaB in cardiovascular disease: diverse. and specific effects of a "general" transcription factor? Cardiovasc. Toxicol. 5, 183–202. 10.1385/ct:5:2:183 16046793

[B17] Kankeu C. Clarke K. Passante E. Huber H. J. (2016). Doxorubicin-induced chronic dilated cardiomyopathy—the apoptosis hypothesis revisited. J. Mol. Med. 95, 239–248. 10.1007/s00109-016-1494-0 27933370

[B18] Kostin S. Rieger M. Dammer S. Hein S. Richter M. Klovekorn W. P. (2003). Gap junction remodeling and altered connexin43 expression in the failing human heart. Mol. Cell. Biochem. 242, 135–144. 10.1023/a:1021154115673 12619876

[B19] Kravchenko V. V. Mathison J. C. Schwamborn K. Mercurio F. Ulevitch R. J. (2003). IKKi/IKKepsilon plays a key role in integrating signals induced by pro-inflammatory stimuli. J. Biol. Chem. 278, 26612–26619. 10.1074/jbc.M303001200 12736252

[B20] Kumar S. Wei C. Thomas C. M. Kim I. K. Seqqat R. Kumar R. (2012). Cardiac-specific genetic inhibition of nuclear factor-κB prevents right ventricular hypertrophy induced by monocrotaline. Am. J. Physiol. Heart Circ. Physiol. 302, H1655–H1666. 10.1152/ajpheart.00756.2011 22245771

[B21] Le Dour C. Macquart C. Sera F. Homma S. Bonne G. Morrow J. P. (2017). Decreased WNT/β-catenin signalling contributes to the pathogenesis of dilated cardiomyopathy caused by mutations in the lamin a/C gene. Hum. Mol. Genet. 26, 333–343. 10.1093/hmg/ddw389 28069793 PMC6075603

[B22] Liu Z. Gan L. Xu Y. Luo D. Ren Q. Wu S. (2017). Melatonin alleviates inflammasome-induced pyroptosis through inhibiting NF-kappaB/GSDMD signal in mice adipose tissue. J. Pineal Res. 63, 632017. 10.1111/jpi.1241 28398673

[B23] Liu Y. Jiang B. Cao Y. Chen W. Yin L. Xu Y. (2020). High expression levels and localization of Sox5 in dilated cardiomyopathy. Mol. Med. Rep. 22, 948–956. 10.3892/mmr.2020.11180 32468049 PMC7339405

[B24] Maier H. J. Schips T. G. Wietelmann A. Krüger M. Brunner C. Sauter M. (2012). Cardiomyocyte-specific IκB kinase (IKK)/NF-κB activation induces reversible inflammatory cardiomyopathy and heart failure. Proc. Natl. Acad. Sci. U. S. A. 109 (29), 11794–11799. 10.1073/pnas.1116584109 22753500 PMC3406816

[B25] Maron B. J. Towbin J. A. Thiene G. Antzelevitch C. Corrado D. Arnett D. (2006). Contemporary definitions and classification of the cardiomyopathies: an American heart association scientific statement from the council on clinical cardiology, heart failure and transplantation committee; quality of Care and outcomes research and functional genomics and translational biology interdisciplinary working groups; and council on epidemiology and prevention. Circulation 113, 1807–1816. 10.1161/CIRCULATIONAHA.106.174287 16567565

[B26] Mazelin L. Panthu B. Nicot A. S. Belotti E. Tintignac L. Teixeira G. (2016). mTOR inactivation in myocardium from infant mice rapidly leads to dilated cardiomyopathy due to translation defects and p53/JNK-mediated apoptosis. J. Mol. Cell. Cardiol. 97, 213–225. 10.1016/j.yjmcc.2016.04.011 27133769

[B27] Mehta L. S. Watson K. E. Barac A. Beckie T. M. Bittner V. Cruz-Flores S. (2018). Cardiovascular disease and breast cancer: Where these entities intersect: A scientific statement from the American heart association. Circulation 137, e30–e66. 10.1161/CIR.0000000000000556 29437116 PMC6722327

[B28] Moser C. V. Kynast K. Baatz K. Russe O. Q. Ferreiros N. Costiuk H. (2011). The protein kinase IKKε is a potential target for the treatment of inflammatory hyperalgesia. J. Immunol. 187, 2617–2625. 10.4049/jimmunol.1004088 21813779

[B29] Patel M. N. Bernard W. G. Milev N. B. Cawthorn W. P. Figg N. Hart D. (2015). Hematopoietic IKBKE limits the chronicity of inflammasome priming and metaflammation. Proc. Natl. Acad. Sci. U. S. A. 112, 506–511. 10.1073/pnas.1414536112 25540417 PMC4299251

[B30] Peters R. T. Liao S. M. Maniatis T. (2000). IKKepsilon is part of a novel PMA-inducible IkappaB kinase complex. Mol. Cell 5, 513–522. 10.1016/s1097-2765(00)80445-1 10882136

[B31] Piek A. Sillje H. H. W. de Boer R. A. (2019). The vicious cycle of arrhythmia and myocardial fibrosis. Eur. J. Heart Fail. 21, 492–494. 10.1002/ejhf.1421 30698320

[B32] Schultheiss H-P. Fairweather D. Caforio A. L. P. Escher F. Hershberger R. E. Lipshultz S. E. (2019). Dilated cardiomyopathy. Nat. Rev. Dis. Prim. 5, 32. 10.1038/s41572-019-0084-1 31073128 PMC7096917

[B33] Shimada T. Kawai T. Takeda K. MatsuMotoM. Inoue J. Tatsumi Y. (1999). IKK-i, a novel lipopolysaccharide-inducible kinase that is related to IkappaB kinases. Int. Immunol. 11, 1357–1362. 10.1093/intimm/11.8.1357 10421793

[B34] Singal P. K. Iliskovic N. (1998). Doxorubicin-induced cardiomyopathy. N. Engl. J. Med. 339, 900–905. 10.1056/NEJM199809243391307 9744975

[B35] Solt L. A. May M. J. (2008). The IkappaB kinase complex: master regulator of NF-kappaB signaling. Immunol. Res. 42, 3–18. 10.1007/s12026-008-8025-1 18626576 PMC2965074

[B36] Vande Walle L. Lamkanfi M. (2016). Pyroptosis. Curr. Biol. 26, R568–R572. 10.1016/j.cub.2016.02.019 27404251

[B37] Verhelst K. Verstrepen L. Carpentier I. Beyaert R. (2013). IκB kinase ε (IKKε): a therapeutic target in inflammation and cancer. Biochem. Pharmacol. 85, 873–880. 10.1016/j.bcp.2013.01.007 23333767 PMC7111187

[B38] Weintraub R. G. Semsarian C. Macdonald P. (2017). Dilated cardiomyopathy. Lancet 390, 400–414. 10.1016/S0140-6736(16)31713-5 28190577

[B39] Wu J. Guo W. Lin S. Z. Wang Z. J. Kan J. T. Chen S. Y. (2016). Gp130-mediated STAT3 activation by S-propargyl-cysteine, an endogenous hydrogen sulfide initiator, prevents doxorubicin-induced cardiotoxicity. Cell Death Dis. 7, e2339. 10.1038/cddis.2016.209 27537522 PMC5108313

[B40] Xia Y. Chen Z. Chen A. Fu M. Dong Z. Hu K. (2017). LCZ696 improves cardiac function via alleviating Drp1-mediated mitochondrial dysfunction in mice with doxorubicin-induced dilated cardiomyopathy. J. Mol. Cell. Cardiol. 108, 138–148. 10.1016/j.yjmcc.2017.06.003 28623750

[B41] Yang Z. Honda T. Ueda K. (2018). vFLIP upregulates IKKε, leading to spindle morphology formation through RelA activation. Virology 522, 106–121. 10.1016/j.virol.2018.07.007 30029010

[B42] Yi W. Xiaolu L. Lining Z. Shen Y. Chengedza S. Feng H. (2013). IKK epsilon kinase is crucial for viral G protein-coupled receptor tumorigenesis. Proc. Natl. Acad. Sci. U. S. A. 110, 11139–11144. 10.1073/pnas.1219829110 23771900 PMC3703966

[B43] Yin H. Guo X. Chen Y. Zeng Y. Mo X. Hong S. (2022). TAB2 deficiency induces dilated cardiomyopathy by promoting RIPK1-dependent apoptosis and necroptosis. J. Clin. Invest. 132, e152297. 10.1172/JCI152297 34990405 PMC8843707

[B44] Zhang J. Tian M. Xia Z. Feng P. (2016). Roles of IκB kinase ε in the innate immune defense and beyond. Virol. Sin. 31, 457–465. 10.1007/s12250-016-3898-y 28063014 PMC8193418

[B45] Zhang Y. Zhang M. Xu W. Chen J. Zhou X. (2017). The long non-coding RNA H19 promotes cardiomyocyte apoptosis in dilated cardiomyopathy. Oncotarget 8, 28588–28594. 10.18632/oncotarget.15544 28430627 PMC5438674

[B46] Zhou Z. Qi J. Zhao J. Lim C. W. Kim J. W. Kim B. (2019). Dual TBK1/IKKɛ inhibitor amlexanox attenuates the severity of hepatotoxin-induced liver fibrosis and biliary fibrosis in mice. J. Cell. Mol. Med. 24, 1383–1398. 10.1111/jcmm.14817 31821710 PMC6991653

