# Supplementary material for: I-κB kinase-ε deficiency improves doxorubicin-induced dilated cardiomyopathy by inhibiting the NF-κB pathway
Source: Front Physiol. 2022 Aug 4;13:934899. doi: 10.3389/fphys.2022.934899 (PMC9386238; doi:10.3389/fphys.2022.934899)
Supplement: Supplementary file 1 [file DataSheet1.pdf]

Supplementary Tabel1. Primers used for quantitative real-time PCR

	Forward	Reverse
ANP	5'-AAGAACCTGCTAGACCACCTGGAG-3'	5'-TGCTTCCTCAGTCTGCTCACTCAG-3'
BNP	5'-GGAAGTCCTAGCCAGTCTCCAGAG-3'	5'-GCCTTGGTCCTTCAAGAGCTGTC-3'
β-MHC	5'-GCAAGACGGTGACTGTGAAGGAG-3'	5'-GGTTGACGGTGACGCAGAAGAG-3'
Acta-1	5'-GCGTGGCTATTCCTTCGTGACC-3'	5'-ACGCTCATTGCCGATGGTGATG-3
IL-1β	5'-TCGCAGCAGCACATCAACAAGAG-3'	R-5'-TGCTCATGTCCTCATCCTGGAAGG-3
TNF-α	5'-ACGGCATGGATCTCAAAGAC-3'	5'-AGATAGCAAATCGGCTGACG-3'
IL-6	5'-ACAACGATGATGCACTTGCAGA-3'	5'-GATGA ATTGGATGGTCTTGGTC-3'
IL-10	5'-GCTCTTACTGACTGGCATGAG-3'	5'-CGCAGCTCTAGGAGCATGTG-3'
TGF-β1	5'-GCAACAATTCCTGGCGTTACCTTG-3'	5'-CAGCCACTGCCGTACA ACTCC-3'
CTGF	5'-CACCGCACAGAACCACCACTC-3'	5'-AATGGCAGGCACAGGTCTTGATG-3'
Collagen 1a1	5'-TGGTCCTGCTGGTCCTGCTG-3'	5'-CTGTCACCTTGTTCGCCTGTCTC-3'
Collagen 3a1	5'-TCTCCTGGTGCTGCTGGTCAC-3'	5'-TCCATGTGGTCCAACTGGTCCTC-3'
GAPDH	5'-ACCACAGTCCATGCCATCAC-3'	5'-TCCACCACCCTGTTGCTGTA-3'

Supplementary Tabel2. heart weight and body weight of mice after Dox-induced

number	Heart weight(mg)	Body weight(g)
WT+Saline1	128	32
WT+Saline2	129	30
WT+Saline3	124	32
WT+Saline4	131	30
WT+Saline5	131	34
WT+Saline6	125	29
IKKε-KO+Saline1	129	34
IKKε-KO+Saline2	121	30
IKKε-KO+Saline3	119	27
IKKε-KO+Saline4	127	29
IKKε-KO+Saline5	124	29
IKKε-KO+Saline6	131	31
WT+Dox1	143	22
WT+Dox2	146	24
WT+Dox3	146	24
WT+Dox4	139	25
WT+Dox5	148	25
WT+Dox6	136	22
IKKε-KO+Dox1	131	26
IKKε-KO+Dox2	130	28
IKKε-KO+Dox3	128	26
IKKε-KO+Dox4	127	26

IKKε-KO+Dox5	132	27
IKKε-KO+Dox6	133	25
